# Electronic cigarettes use among teenagers in Tunisia

**DOI:** 10.1192/j.eurpsy.2021.2173

**Published:** 2021-08-13

**Authors:** R. Maalej, Y. Zgueb, A. Aissa, U. Ouali, F. Nacef

**Affiliations:** 1 Psychiatry A, Razi Hospital, Manouba, Tunisia; 2 Psychiatry A Department, Razi Hospital, Manouba, Tunisia

**Keywords:** Electronic Nicotine Delivery Systems, adolescent

## Abstract

**Introduction:**

Electronic cigarettes (e-cigarettes) are battery-powered devices developed with the goal of mimicking the action of smoking. Their use has increased over the past years.

**Objectives:**

The aim of this study was to evaluate the prevalence of the use of e-cigarettes among teenagers in high school and to examine the predictor factors.

**Methods:**

A survey was conducted with a sample of 234 students in Mohamed Ali high school in Sfax, a town in the south of Tunisia, in February 2020. We estimated e-cigarette prevalence among adolescents and the predictor factors of vaping.

**Results:**

Among high school students aged 15 to 20, 58,8% have ever used e-cigarette, 38,3% had done so within the previous 30 days and 20,5 % were regular users of vapes. The mean age of e-cigarette users was 16,59 +/- 0,908 years old, 83,3% of e-cigarettes users were males. Male gender, high socio-economic level, practicing leisure activities, smoking cigarettes and drinking alcohol were associated with regular use of e-cigarettes. Overall, 51, 6% of never smoking students reported ever use of e-cigarettes. The main reason for initiating e-cigarettes use was curiosity (65%).

**Conclusions:**

Our findings showed the significant use of e-cigarettes among high school students thus it would be interesting to provide adolescents with information about the use of e-cigarettes and other tobacco products.

**Disclosure:**

No significant relationships.

